# AXIN2 Reduces the Survival of Porcine Induced Pluripotent Stem Cells (piPSCs)

**DOI:** 10.3390/ijms222312954

**Published:** 2021-11-30

**Authors:** Rui Zhang, Shuai Yu, Qiaoyan Shen, Wenxu Zhao, Juqing Zhang, Xiaolong Wu, Zhenshuo Zhu, Xiaojie Wu, Na Li, Sha Peng, Jinlian Hua

**Affiliations:** Shaanxi Centre of Stem Cells Engineering & Technology, College of Veterinary Medicine, Northwest A&F University, Yangling 712100, China; zrui@nwafu.edu.cn (R.Z.); yus2017@nwafu.edu.cn (S.Y.); sqy@nwafu.edu.cn (Q.S.); zhaowx@nwafu.edu.cn (W.Z.); 2013015382@nwafu.edu.cn (J.Z.); 2018050593@nwafu.edu.cn (X.W.); 2016060217@nwafu.edu.cn (Z.Z.); wuxiaojie@nwafu.edu.cn (X.W.); lina2017@nwsuaf.edu.cn (N.L.); pengshacxh@nwafu.edu.cn (S.P.)

**Keywords:** piPSC, pluripotency, WNT, AXIN, apoptosis

## Abstract

The establishment of porcine pluripotent stem cells (piPSCs) is critical but remains challenging. All piPSCs are extremely sensitive to minor perturbations of culture conditions and signaling network. Inhibitors, such as CHIR99021 and XAV939 targeting the WNT signaling pathway, have been added in a culture medium to modify the cell regulatory network. However, potential side effects of inhibitors could confine the pluripotency and practicability of piPSCs. This study aimed to investigate the roles of AXIN, one component of the WNT pathway in piPSCs. Here, porcine AXIN1 and AXIN2 genes were knocked-down or overexpressed. Digital RNA-seq was performed to explore the mechanism of cell proliferation and apoptosis. We found that (1) overexpression of the porcine AXIN2 gene significantly reduced survival and negatively impacted the pluripotency of piPSCs, and (2) knockdown of AXIN2, a negative effector of the WNT signaling pathway, enhanced the expression of genes involved in cell cycle but reduced the expression of genes related to cell differentiation, death, and apoptosis.

## 1. Introduction

The Wnt/β-catenin signaling pathway is critical for embryonic development and has a function in many cellular processes, including tumorigenesis, cell proliferation, differentiation, pluripotency, migration, and apoptosis [1]. CHIR99021, an inhibitor of glycogen synthase kinase 3 (GSK3), is wildly used in induced pluripotent stem cell (iPSC) culture condition to activate the Wnt/β-catenin signaling pathway for the maintenance of pluripotency [2]. However, the use of chemical inhibitors can compromise cell epigenetic and genomic stability [3,4]. As a result, it will be beneficial to find ways to activate the Wnt/β-catenin signaling pathway without using inhibitors.

β-catenin (CTNNB1) is the effector molecule of the Wnt signaling pathway [5]. CTNNB1 is degraded by a destruction complex that includes AXIN1/2, adenomatous polyposis coli (APC), GSK3β, and casein kinase 1 (CK1) [6]. CTNNB1 is not only in favor of the self-renewal of pluripotent state of stem cells, but also regulates the reprogramming process. Three groups independently studied CTNNB1 in mouse embryonic stem cells (mESCs) and found that CTNNB1 is indispensable for mESC self-renewal [7,8,9]. In addition, CTNNB1 has been linked to the maintenance of the pluripotent state in human and mouse ESCs [10]. Notably, CTNNB1 interacts with reprogramming factors KLF4, OCT4, and SOX2, further enhancing the expression of pluripotency circuitry genes [11]. Wnt/β-catenin signaling has also been shown to enhance the induction of pluripotency in somatic cells through viral transduction or cell fusion [12].

AXINs provide a crucial scaffold for the CTNNB1 destruction complex [13]. Upon receptor activation by Wnt ligands, AXIN1 and/or AXIN2 are recruited to the phosphorylated tail of low-density lipoprotein receptor-related protein (LRP), and the re-localization inhibits CTNNB1 ubiquitination, resulting in CTNNB1 accumulation and translocation to the nucleus [13,14]. Thus, AXIN1 and/or AXIN2 are pivotal in controlling canonical Wnt signaling [14,15,16,17]. 

Pigs are more similar to humans than rodents in organ size, physiology, and anatomy, and thus, autologous and/or homologous transplantation using pig iPS cell derivatives represents a superior model for regenerative medicine [18,19]. Moreover, the maintenance of the pluripotent state for many reported piPSCs was dependent on the regulation of the Wnt/β-catenin pathway [20]. However, as the key component of WNT signaling, the role of AXIN in piPSCs was unclear. 

Here, we confirmed AXIN2 worked as a negative effector for the Wnt/β-catenin signaling pathway in piPSCs, and a reduced level of AXIN2 improved the ability of piPSCs to proliferate and resist apoptosis and, importantly, replaced the need of CHIR99021. Our data not only suggested that AXIN2 possesses a stronger effect on the piPSCs than AXIN1, but explained the potential mechanism underlying this process.

## 2. Results

### 2.1. The Absence of CHIR99021 Affected the Pluripotency and Proliferation of piPSCs

**Figure 1:**
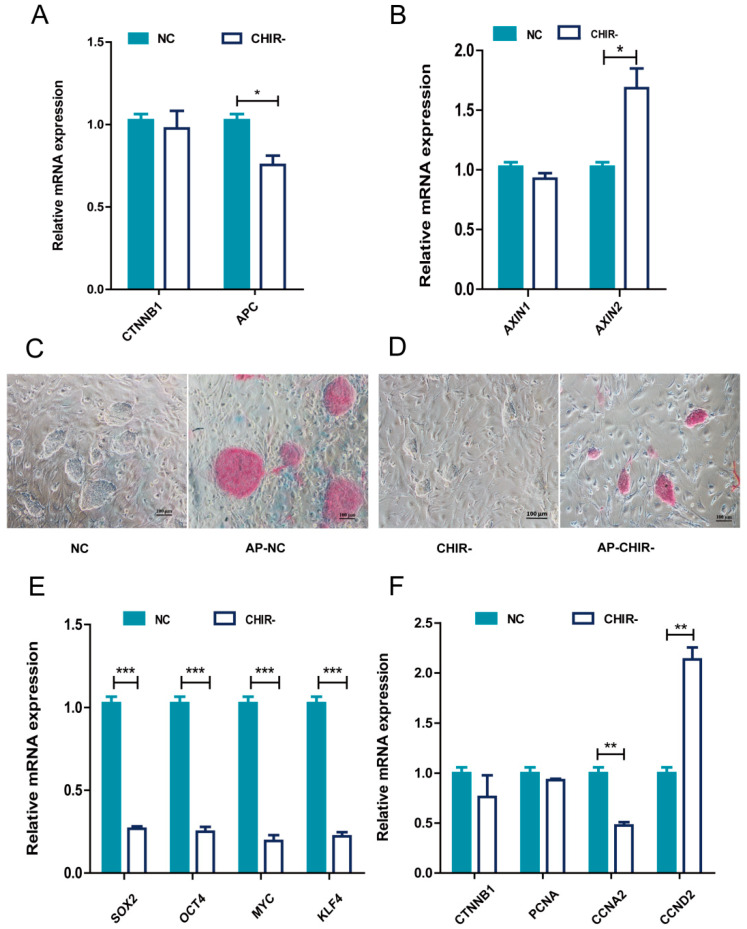
CHIR99021 is crucial to maintain the pluripotency and proliferation of piPSCs. (**A**)qPCR analysis of the expression levels of *CTNNB1* and *APC* in the NC and CHIR- groups, (**B**) qPCR analysis of the expression levels of AXIN1 and AXIN2 in the NC and CHIR- groups, (**C**,**D**) Representative image of colonies and AP staining after 5 days of clonal growth of piPSCs in the control (NC) and removal of CHIR99021 (CHIR-) conditions, (**E**) qPCR analysis of the expression levels of endogenous *OCT4*, *SOX2*, *KLF4*, and *MYC* in the NC and CHIR- groups (**F**) qPCR analysis of the expression levels of *CCND1*, *CCND2*, *PCNA*, and *CCNA2* in NC and CHIR- groups; * represents *p* < 0.05, ** represents *p* < 0.001, and *** represents *p* < 0.0001.

To investigate whether WNT signals are essential for the survival of piPSCs, we withdrew the WNT activator CHIR99021 for three passages so that its effect could fade away. We tested the expression levels of *CTNNB1*, *APC*, AXIN1, and AXIN2, which are key components of the WNT cascade. The Quantitative Real-time PCR (qPCR) results revealed a dropped amount of *APC* and an elevated AXIN2 without CHIR99021 (Figure 1A,B), which confirmed that the WNT signaling pathway was modulated by CHIR99021. To gain a deeper understanding of the cellular impacts, we further investigated the effects on the pluripotency and proliferation of piPSCs. The results showed that the Alkaline Phosphatase (AP) staining was weaker, and the piPSC colony became smaller after CHIR99021 was withdrawn (Figure 1C,D). Further qPCR analysis of pluripotent genes revealed a significant decrease in the expression of endogenous *OCT4*, *SOX2*, *KLF4*, and *MYC* (Figure 1E), indicating a negative impact on pluripotency by the removal of the WNT activator. Then, we explored whether the smaller colonies observed upon CHIR99021 withdrawal were related to a decrease in cell proliferation by testing cell cycle-related genes. Although the expression of *CCND2* was up-regulated, the decrease of *CCNA2*, *CCND1*, and the smaller colony size implicated a positive correlation between WNT signaling and the cell cycle of piPSCs. These data suggested that CHIR99021 could maintain the pluripotency and proliferation of piPSCs via stimulating the WNT signaling pathway.

### 2.2. AXIN2 Had a Stronger Influence on the Pluripotency of piPSCs Than AXIN1

**Figure 2:**
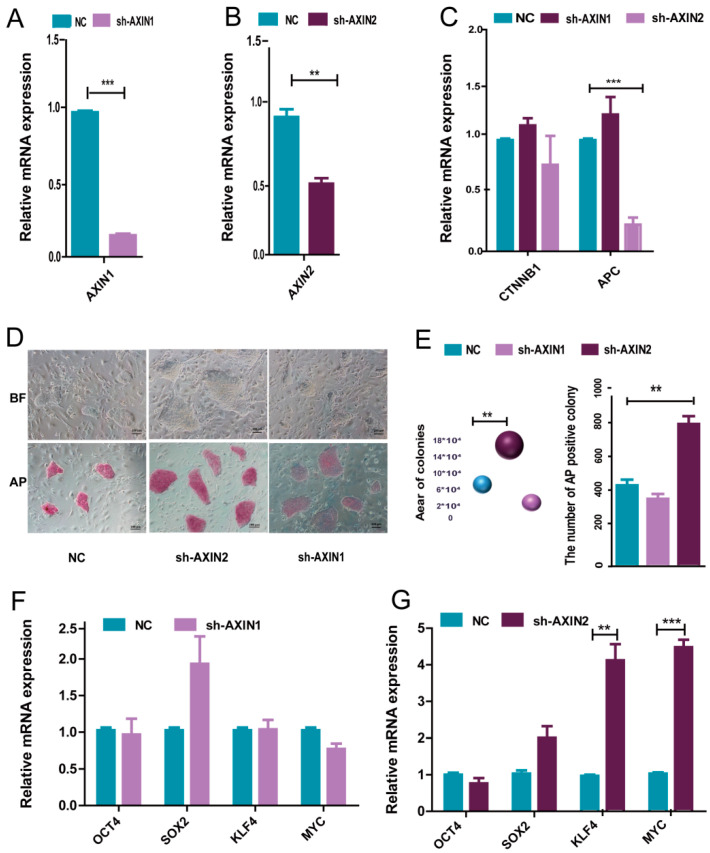
Knockdown of AXIN2 showed higher pluripotency in piPSCs. (**A**) qPCR analysis of the expression levels of AXIN1 in the control (NC) and knockdown AXIN1 (sh-AXIN1) groups; (**B**) qPCR analysis of the expression levels of AXIN2 in the control (NC) and knockdown AXIN2 (sh-AXIN2) groups; (**C**) qPCR analysis of the expression levels of and *APC* in NC, sh-AXIN1, and sh-AXIN2 groups; (**D**) representative image of colonies and AP staining after 5 days of clonal growth in the NC, sh-AXIN1, and sh-AXIN2 groups; the scale bar represents 100 μm; (**E**) the area of NC, sh-AXIN1, and shAXIN2 colonies; the quantities of AP positive colonies in the NC, sh-AXIN1, and sh-AXIN2 groups; (**F**) qPCR analysis of endogenous *OCT4*, *SOX2*, *KLF4*, and *MYC* in NC and sh-AXIN1 groups; (**G**) qPCR analysis of endogenous *OCT4*, *SOX2*, *KLF4*, and *MYC* in NC and sh-AXIN2 groups; ** represents *p* < 0.001, and *** represents *p* < 0.0001.

As AXINs provide a crucial scaffold for WNT signaling and an increased amount of AXIN2 was observed in the CHIR- group, we further investigated the role of AXIN1 and AXIN2 in piPSCs. We first generated AXIN1 and AXIN2 knockdown (sh-AXIN1/AXIN2) piPSC lines using a lentiviral vector. The qPCR analysis showed that in sh-AXIN1 piPSCs lines, the expression of AXIN1 was about 80% reduced compared with that of the NC group and that AXIN2 in sh-AXIN2 piPSCs lines was about 58% reduced than that of the NC group (Figure 2A,B). A western blot analysis also showed that AXIN1 and AXIN2 were knockdown (Supplementary Figure S1A,B). There was also a significant decrease of *APC* in the sh-AXIN2 group but no fluctuation in the sh-AXIN1 group (Figure 2C). This result indicates that AXIN2, but not AXIN1, positively influences the expression of *APC*.

AP staining was then used to determine the pluripotency of sh-AXIN1 and sh-AXIN2 piPSCs. The area of the colonies was measured, and the number of AP positive colonies was counted. The results showed that the sh-AXIN2 piPSCs formed bigger colonies with stronger AP staining, while sh-AXIN1 piPSCs showed no significant change compared to the NC group (Figure 2D,E). This result indicated an improved pluripotency in sh-AXIN2 piPSCs. Then, we examined the expression level of endogenous *OCT4*, *SOX2*, *KLF4*, and *MYC* in sh-AXIN2, sh-AXIN1, and NC groups by qPCR, and the results revealed significant up-regulated *KLF4* and *MYC* in sh-AXIN2 but no evident change in the sh-AXIN1 group (Figure 2F,G). This result indicated that AXIN2 could affect the pluripotent state of piPSCs.

**Figure 3:**
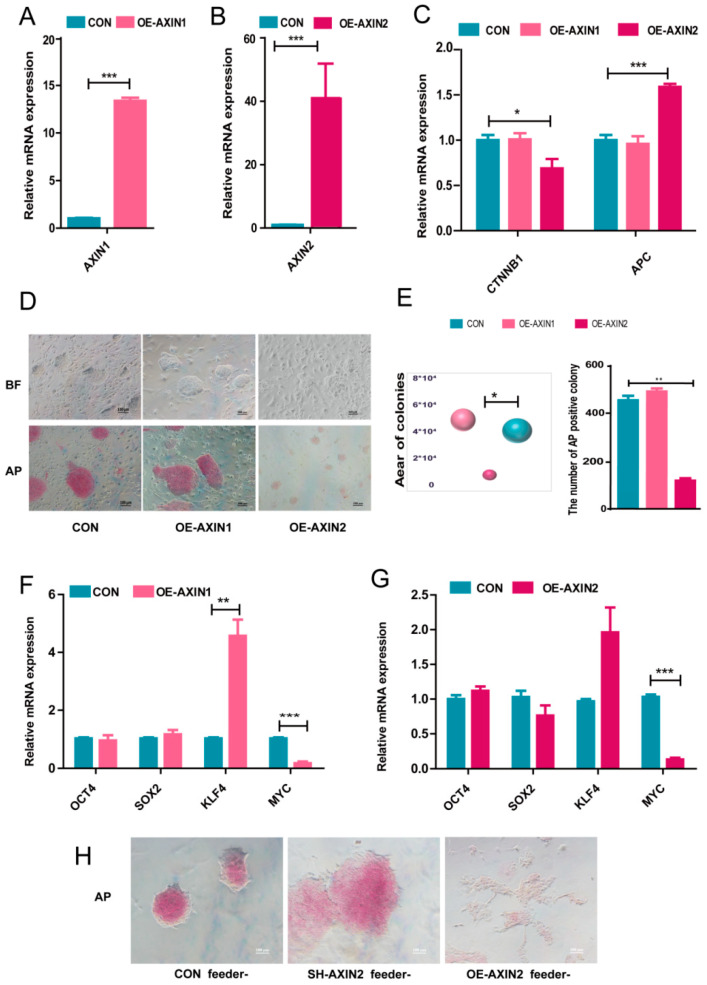
Overexpression of AXIN1 and AXIN2 in piPSCs. (**A**) qPCR analysis of the expression levels of AXIN1 in the control (CON) and overexpressing AXIN1 (OE-AXIN1) groups; (**B**) qPCR analysis of AXIN2 in the control (CON) and overexpressing AXIN2 (OE-AXIN2) groups; (**C**) qPCR analysis of and *APC* in the CON, OE-AXIN1, and OE-AXIN2 groups; (**D**) representative image of bright and AP positive colonies after 5 days of clonal growth in the CON, OE-AXIN1, and OE-AXIN2 groups; the scale bar represents 100 μm; (**E**) the area of CON, OE-AXIN1, and OE-AXIN2 colonies; the quantities of AP positive colonies in the CON, OE-AXIN1, and OE-AXIN2 groups; (**F**) qPCR analysis of endogenous *OCT4*, *SOX2*, *KLF4*, and *MYC* in NC and OE-AXIN1; (**G**) qPCR analysis of endogenous *OCT4*, *SOX2*, *KLF4*, and *MYC* in the CON and OE-AXIN2 groups; (**H**) representative image of AP staining after 5 days of clonal growth without feeder in the CON, sh-AXIN2, and OE-AXIN2 groups; the scale bar represents 100 μm. * represents *p* < 0.05, ** represents *p* < 0.001, *** represents *p* < 0.0001.

We next constructed AXIN1 overexpression (OE-AXIN1) and AXIN2 overexpression (OE-AXIN2) cell lines to further investigate the role of AXIN1 and AXIN2 in piPSCs. qPCR showed that the expression of AXIN1 in OE-AXIN1 piPSCs was significantly higher than that of the CON group, and in OE-AXIN2 piPSCs lines, the expression of AXIN2 was about 80 times higher than the NC group (Figure 3A,B). A western blotting analysis showed that AXIN1 and AXIN2 were overexpressed successfully (Supplementary Figure S1A,B). Moreover, the expression level of *APC* was also up-regulated in the OE-AXIN2 group (Figure 3C), which provides a shred of evidence to our previous conclusion: *APC* gene expression is positively regulated by AXIN2 but not AXIN1. The level also decreased in the OE-AXIN2 group (Figure 3C). Then, the pluripotency of OE-AXIN1 and OE-AXIN2 was examined with AP staining. The area of the colonies was measured, and the number of AP positive colonies was counted. The result showed that the AP staining was much weaker in OE-AXIN2 than that of the CON group, while OE-AXIN1 piPSCs showed similar staining as the NC group (Figure 3D,E). The qPCR results revealed the mRNA level of *MYC* was significantly down-regulated in the OE-AXIN2 and OE-AXIN1 groups, and the RNA level of *KLF4* was up-regulated in the OE-AXIN1 group (Figure 3F,G). Taken together, our results demonstrated that AXIN2 exerts a stronger influence on the pluripotency of piPSCs than AXIN1.

We previously found [21] that feeder cells could influence the pluripotent state of piPSCs. Thus, because of the significant changes in pluripotency in the sh-AXIN2 and OE-AXIN2 groups, we cultured CON, sh-AXIN2, and OE-AXIN2 cell lines without the feeder. The AP staining showed that OE-AXIN2 piPSCs with the weakest AP staining started to differentiate and lost the ability to yield colonies, while sh-AXIN2 piPSCs formed bigger colonies with stronger AP staining than that of the CON group (Figure 3H). Thus, overexpression of AXIN2 significantly impaired pluripotency of piPSCs.

### 2.3. AXIN2 Regulates the Proliferation of piPSCs via CCND1

**Figure 4:**
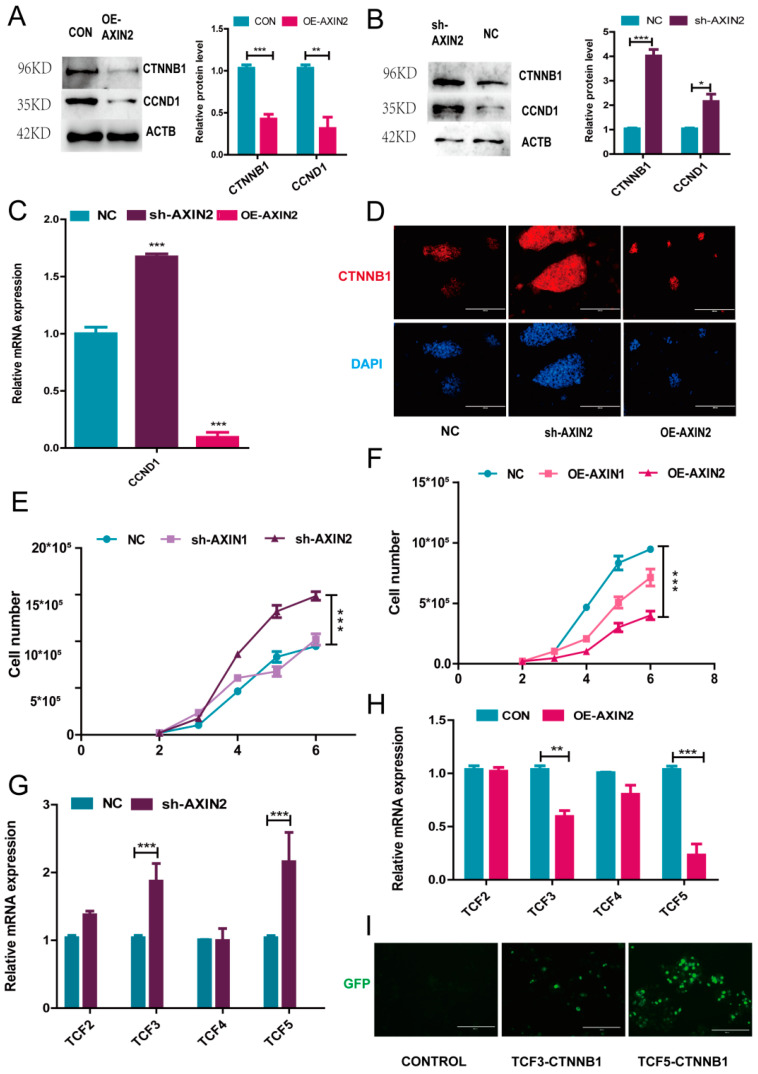
AXIN2 affects cell proliferation through *CCND1*. (**A**) Western blot analysis of CTNNB1 and CCND1 in the CON and OE-AXIN2 groups; (**B**) western blot analysis of CTNNB1 and CCDN1 in the NC and sh-AXIN2 groups; (**C**) qPCR analysis of *CCND1* in the NC, sh-AXIN2, and OE-AXIN2 groups; (**D**) representative image of CTNNB1 immunofluorescence in the NC, sh-AXIN1, and OE-AXIN2 groups; the scale bar represents 200 μm; (**E**) cell growth curve of NC, sh-AXIN1, and sh-AXIN2; *** represents *p* < 0.0001; (**F**) cell growth curve of CON, OE-AXIN1, and OE-AXIN2 groups; *** represents *p* < 0.0001; (**G**) qPCR analysis of *TCF2*, *TCF3*, *TCF4*, and *TCF5* in the NC and sh-AXIN2 groups; (**H**) qPCR analysis of *TCF2*, *TCF3*, *TCF4*, and *TCF5* in the CON and OE-AXIN2 groups; (**I**) representative images of BiFC analysis in CONTROL, TCF3-CTNNB1, and TCF5-CTNNB1 293T cells; the scale bar represents 200 μm. * represents *p* < 0.05, ** represents *p* < 0.001, and *** represents *p* < 0.0001.

A previous study had revealed that AXIN could affect the amount of CTNNB1 in the nucleus, which regulates WNT-downstream genes [1]. We further analyzed the mRNA and protein level of CTNNB1. While OE-AXIN2 significantly decreased the *CTNNB1* mRNA level (Figure 3C), western blotting confirmed an increased CTNNB1 protein level in sh-AXIN2 and a decreased level in OE-AXIN2 groups, respectively (Figure 4A,B). Immunofluorescence also showed that CTNNB1 could hardly be detected in the cell nucleus in the OE-AXIN2 group, while in the sh-AXIN2 group, CTNNB1 was profoundly distributed in cells compared with the NC group (Figure 4D). These results confirmed a negative correlation between the AXIN2 level and the CTNNB1 expression.

At the same time, the piPSC colony morphology change induced by the variation of AXIN2 level intrigued us to form the hypothesis that the altered cell proliferation may be one of the underlying mechanisms. The cell growth curve showed that the cell proliferation increased in the sh-AXIN2 group, while in the OE-AXIN2 group, the proliferation significantly decreased (Figure 4E,F). On the other hand, sh-AXIN1 showed no significant change, while OE-AXIN1 moderately decreased cell proliferation (Figure 4A,B). Moreover, the *CCND1* expression level showed a similar trend as *CTNNB1* after the changing of the AXIN2 expression (Figure 4C). Thus, AXIN2 activity is negatively correlated with the CTNNB1 and CCND1 levels. These results indicated that AXIN2 exerts an obvious stronger effect on piPSCs pluripotency than AXIN1, and AXIN2 suppresses piPSC proliferation by inhibiting the CTNNB1 regulated expression of CCND1.

A previous study showed that the nuclear function of CTNNB1 depends on a transcription factor T-cell factor (TCF) and a lymphocyte-enhancing factor (LEF) [22,23]. Accumulated CTNNB1 is translocated into the nucleus and binds to the TCF/LEF to promote the expression of downstream genes *CCND1* and *MYC* [22]. We further analyzed *TCF2*, *TCF3*, *TCF4*, and *TCF5* by qPCR, and the results showed that only the expression levels of *TCF3* and *TCF5* were changed by AXIN2 knockdown (Figure 4G). To identify whether CTNNB1 could team up with TCF3 or TCF5 in piPSCs, we conducted a Bimolecular Fluorescence Complementation (BiFC) analysis. The BiFC results revealed that both TCF3 and TCF5 proteins bind to CTNNB1 (Figure 4I). These results indicate that AXIN2 exerts its effect on the piPSC proliferation through the AXIN2/CTNNB1/TCF3,5/CCND1 axis.

To further verify the axis, we used the inhibitor XAV939 to repress CTNNB1 (Supplementary Figure S2A) and examine cell proliferation and cell viability. First, we tested the expression levels of and *APC* by qPCR, and the results revealed a decreased amount of *CTNNB1* and *APC* in the sh-AXIN2 XAV939 group (Supplementary Figure S2B), which confirmed that the expression of *CTNNB1* was blocked. To gain a deeper understanding of the cellular influences, we analyzed the expression levels of *TCF3* and *TCF5*, and the qPCR analysis revealed that *TCF3* and *TCF5* were down-regulated when CTNNB1 was inhibited (Supplementary Figure S2C). We further investigated the effects on proliferation and apoptosis. The qPCR analysis of proliferation-related genes *CCND1* and *CCND2* indicated a negative impact on proliferation by the repression of *CTNNB1* (Supplementary Figure S2D), while the qPCR results indicated that the anti-apoptotic ability was slightly influenced by CTNNB1 (Supplementary Figure S2E). Taken together, our data indicated that *CTNNB1* affects the expression of *TCF3* and *TCF5* and regulates proliferation.

### 2.4. AXIN2 Knockdown Enhanced Anti-Apoptotic Ability of piPSCs through Inhibiting Caspase Family and BAX

**Figure 5:**
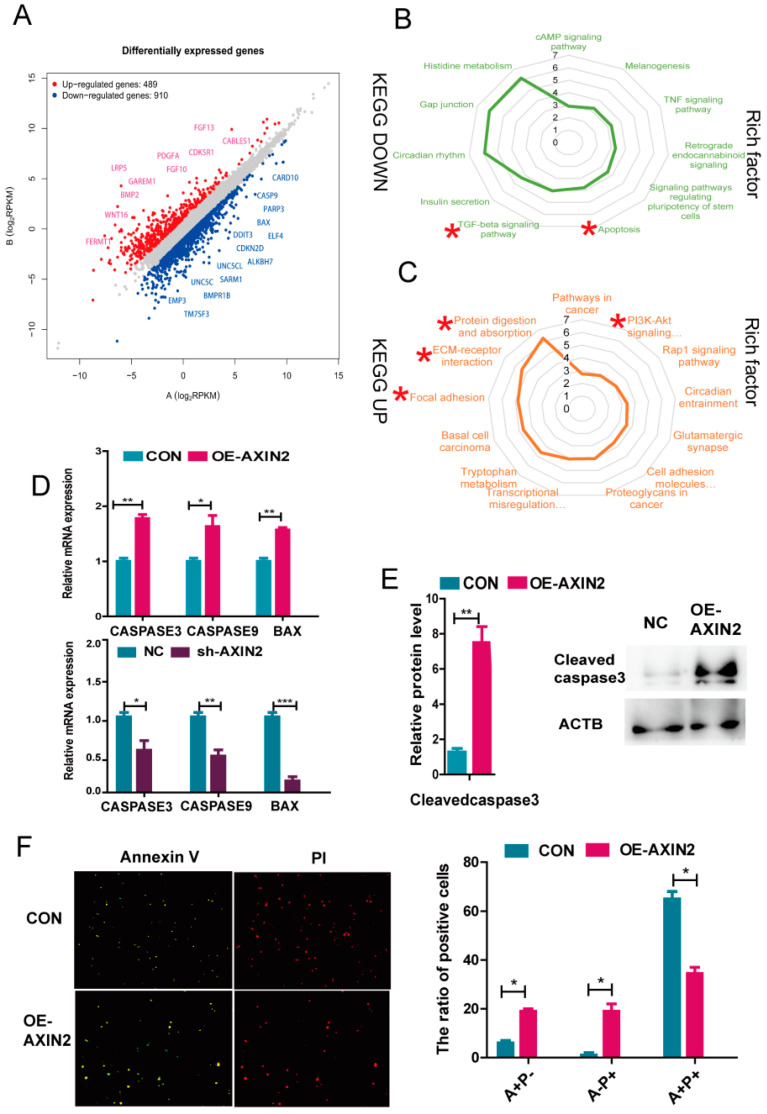
AXIN2 affects the survival of piPSCs through the caspase family. (**A**) Transcriptional changes of the NC and sh-AXIN2 piPSC groups; (**B**) KEGG enrichment of down-differentially expressed genes in sh-AXIN2 cell lines; (**C**) KEGG enrichment of up-differentially expressed genes in sh-AXIN2 cell lines; (**D**) qPCR analysis of *CASPASE3*, *CASPASE 9*, and *BAX* in NC, sh-AXIN2, and CON, OE-AXIN2 groups; * represents *p* < 0.05, ** represents *p* < 0.001, and *** represents *p* < 0.0001; (**E**) western blot analysis of Cleaved caspase3 in the NC and OE-AXIN2 groups; (**F**) representative images of piPSCs stained with PI (P) and Annexin V (A) after 5 d of culture in the Left; NC and OE-AXIN2 groups. A+/P+: non-viable apoptotic cell or necrotic cells; A−/P+: mechanic injury; A+/P−: viable apoptotic cell. The quantitative analysis is shown on the right. *n* = 3 independent experiments. The scale bar represents 400 µm; Right, Ratio of positive cell of PI (P) and Annexin V (A) staining; * represents *p* < 0.05.

We attempted to gain insights into the molecular consequences triggered in response to the knockdown of AXIN2. RNA sequencing was performed on these two cell lines after 5 days of culture. Transcriptional changes in both directions were observed after the knockdown of AXIN2 (489 up vs. 910 down), suggesting that the knockdown of AXIN2 mainly promoted the expression of genes regulating cell growth and improving the survival of piPSCs (Figure 5A). To further investigate this, we applied a KEGG analysis for the up-regulated and down-regulated genes. The list of genes up-regulated in the sh-AXIN2 group uncovered the enrichment in KEGG pathways, including protein digestion and absorption, the PI3K-Akt signaling pathway, focal adhesion, ECM−receptor interaction, and cell adhesion molecules (CAMs). On the other hand, the list of genes down-regulated in sh-AXIN2 cells identified the enriched KEGG pathways, including apoptosis andTGF-beta signaling pathways (Figure 5B,C).

We further tested if apoptosis might have played a role on the colony size. In a previous study, a reduced expression level of AXIN2 in embryos decreased apoptosis by up-regulating *BCL2* [22,24]. However, *BCL2* was not significantly influenced by AXIN2 in this study. The qPCR analysis further found that the expression levels of pro-apoptotic protein *BAX*, *CASPASE3*, and *CASPASE9* significantly decreased in sh-AXIN2 but increased in OE-AXIN2 groups (Figure 5D). Western blotting analysis further demonstrated that the expression level of Cleaved caspase3 in the OE-AXIN2 group significantly increased compared to the CON group, further supporting that more cell apoptosis occurred in the OE-AXIN2 group (Figure 5E). We further evaluated the apoptosis of piPSCs after overexpression of AXIN2 by using PI (P) and Annexin V (A) staining. The results revealed that overexpression of AXIN2 significantly promoted the apoptosis of piPSCs (Figure 5F). Taken together, our data indicated that AXIN2 affects *CASPASE3*, *CASPASE9*, and *BAX*, but not *BCL2*, levels to determine the survival of piPSCs.

### 2.5. piPSCs with Reduced Expression of AXIN2 Maintained Self-Renewal without CHIR99021

**Figure 6:**
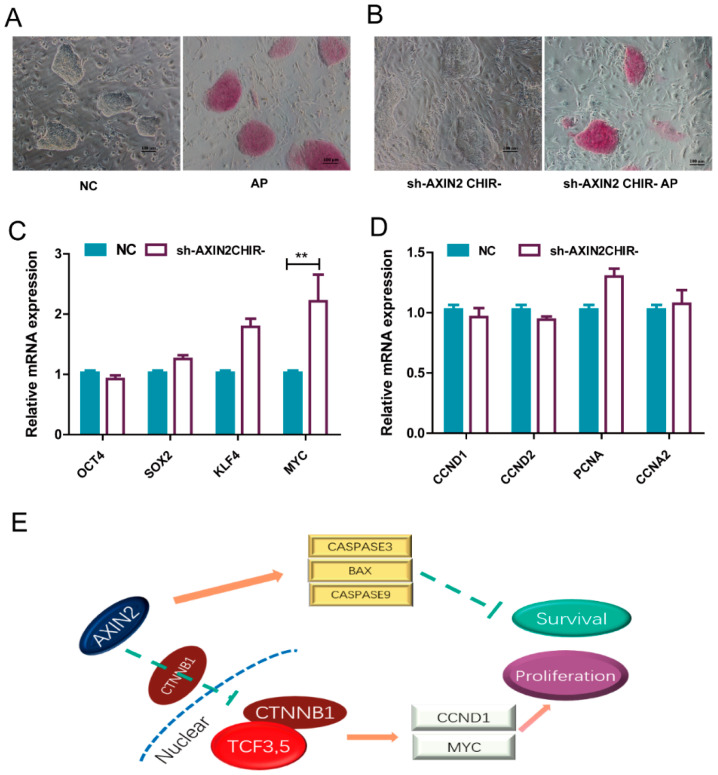
sh-AXIN2 could maintain the proliferation and pluripotency of piPSCs without CHIR99021. (**A**) Representative image of bright field and AP staining in the NC group with CHIR99021; the scale bar represents 100 μm; (**B**) representative image of bright field and AP staining in sh-AXIN2 group withdout CHIR99021; the scale bar represents 100 μm; (**C**) qPCR analysis of endogenous *OCT4*, *SOX2*, *KLF4*, and *MYC* in NC, sh-AXIN2CHIR- groups; ** represents *p* < 0.001; (**D**) qPCR analysis *CCND1, CCND2, PCNA*, and *CCNA2* in NC, sh-AXIN2CHIR- groups; (**E**) diagram of the function of AXIN2 in piPSCs; The arrow in the color of brown means promotion, and in the color of green means suppression.

To verify whether the activated WNT signaling upon AXIN2 knockdown was enough for piPSCs to self-renew, we withdrew CHIR99021 in a culture medium for three passages. There was no significant change to the colony morphology (Figure 6A,B). We further investigated the potential on pluripotency and proliferation of piPSCs. AP staining was used to preliminarily evaluate the pluripotency of piPSCs. We found that the AP staining was similar to the group cultured in the control medium, and qPCR analysis revealed an elevated level of *MYC* (Figure 6C). The cell cycle-related gene expression levels showed no change (Figure 6D). Thus, piPSCs with the AXIN2 knockdown could maintain self-renewal without CHIR99021.

In conclusions, we have demonstrated that AXIN2, but not AXIN1, plays a major role for the degradation of CTNNB1 to restrain piPSC proliferation. Meanwhile, AXIN2 positively regulates the level of the CASPASE family and BAX proteins, resulting in increased cell apoptosis in piPSCs (Figure 6E).

## 3. Discussion

In general, our study revealed that AXIN2 participated in the WNT signaling pathway in piPSCs, and increased expression of AXIN2 counteracted the maintenance of the proliferation and pluripotency of piPSCs. This was, surprisingly, not consistent with the previous study, which indicated XAV939, known as a stabilizer of AXIN1 and AXIN2, could enhance the pluripotency of piPSCs [13]. Moreover, knockdown of AXIN2 could enhance the expression of genes involved in the cell cycle, such as *CCND1*, and reduced the expression of genes related to cell differentiation, cell death, and apoptosis, thus maintaining the pluripotency and survival of piPSCs.

The WNT signaling pathway plays an important role in pluripotency and differentiation, and the AXIN family promotes CTNNB1 ubiquitination and CTNNB1 degradation. However, it was still unknown via which *AXIN* isoform they were promoted through. In this study, we found that AXIN2, but not AXIN1, could regulate the expression level of *KLF4* and *MYC* and maintain piPSCs self-renewal without CHIR99021. The result is consistent with the studies of mouse iPSCs [16,25].

The endogenous *OCT4*, which is the central factor of pluripotency [20], was not activated in piPSCs. Plenty of studies tried to boost the expression of *OCT4*. It was found that the increase in the level of regulated the activity of *OCT4* [11,26,27] and enhanced pluripotency. As CTNNB1 accumulated when AXIN2 was knocked-down, we also examined the effect of on the *OCT4* promoter activity by dual-luciferase reporter assay; however, the negative outcome disappointed us. Until now, the inactivity of *OCT4* was still a mystery of piPSCs [20].

Another huge drawback of the present piPSCs is that it was still hard to contribute to efficient chimeras and was extremely sensitive to perturbation in culture conditions. Previous studies showed that overexpression of *BCL2* enhanced piPSCs’ survival under stress and contributed to chimera formation, and this effect was mediated mainly by the increased resistance to apoptosis [28,29,30]. In our study, we also confirmed that the caspase family *CASPASE3*, *CASPASE9*, and apoptotic protein BAX were down-regulated after AXIN2 knockdown. We propose here that sh-AXIIN2 piPSCs exhibit a greater contribution in chimera formation and will further test this in the near future.

Previous studies showed that the AXIN family may take part in the regulation of other signaling pathways, such as the TGFβ pathway, which is also a core signaling pathway for the pluripotency maintenance of piPSCs [31,32]. AXIN activates TGF-beta signaling by forming a multimeric complex, consisting of Smad7 and ubiquitin E3 ligase, Arkadia [33]. However, we did not perform a functional study by using sh-AXIN2 piPSCs cultured without CHIR99021. Our results inspired us to find a new way to replace the use of SB431542, an inhibitor to the TGFβ signaling pathway, because the list of genes down-regulated in the sh-AXIN2 group revealed enrichment of the TGF−beta pathways. As we demonstrated previously, the use of small molecule inhibitors could impair the differentiation potential of stem cells [3]. Our results here suggested a possibility of a simplified culture medium for piPSCs, which would be one important future direction on piPSCs. There might be better means to regulate the expression of relevant genes to maintain pluripotent network than using chemical inhibitors.

## 4. Methods

### 4.1. Cell Culture

The piPSCs constructed by [34] were used in this study. The piPSCs were cultured on a feeder maintained in LB2i medium [35], which consisted of DMEM (Hyclone) supplemented with 15% FBS (VIS), 10 ng/mL LIF (14890-HNAE, Sino Biological, Beijing, China), 10 ng/mL bFGF (10014-HNAE, Sino Biological, Beijing, China), 0.1 mmol/L NEAA, 1 mmol/L L-glutaMAX, 0.1 mmol/L β-mercaptoethanol (M3148, Sigma-Aldrich, Shanghai, China), 3 µmol/L CHIR99021 (HY-10182, MCE, Beijing, China), 2 µmol/L SB431542 (S1067, Selleck, Shanghai, China), 4 µg/mL Doxycycline (D9891, Sigma-Aldrich, Shanghai, China), and 100 µg/mL streptomycin, 100 units/mL penicillin. The piPSCs were passaged using TrypLE™ Select (12563-029, Invitrogen, Shanghai, Beijing) into a single cell at a ratio of 1:50 every 5 or 6 days.

### 4.2. Plasmid and Cloning

To investigate the function of AXIN, the genes, AXIN1 and AXIN2, were PCR-amplified from piPSCs and then were subcloned into EF1-3FLAG-MCS-T2A-puromycin Lentiviral vectors [30].

In this study, two shRNA vectors of both *Axin1* and *Axin2* were designed by using the online shRNA design tool from Invitrogen (http://rnaidesigner.thermofisher.com/rnaiexpress on 8 October 2019) and subcloned into a pCDH-U6-MCS-EF1-GFP-T2A-puromycin vector [29]. The potential off-target sites were filtered out using BLAST, based on the porcine genome. The shRNA vectors of AXIN1 or AXIN2 were infected in piPSCs, and the knockdown efficiencies 80% and 57%, respectively, were used in this study.

### 4.3. Lentivirus Packaging and Transduction

HEK293T cells used in this study were cultured in DMEM supplemented with 10% FBS, 1 mM L-glutaMAX (A1286001, ThermoFisher Shanghai, China), 0.1 mM (11140050, ThermoFisher, Shanghai, China), 0.1 mM β-mercaptoethanol (444203, Sigma-Aldrich, Shanghai, China), 100 µg/mL of streptomycin, and 100 U/mL of penicillin.

HEK293T cells were seeded onto a 6-well plate and grown to 70% or 80% confluence [36]. Then, the package vector and lentivirus backbone (psPAX2 and pVSV-G) were transfected into HEK293T cells using PEI (polyethyleneimine, Sigma) according to the manufacturer’s instructions.

Then, the medium containing viral particles was obtained from each individual and filtered through a 0.45 µm filter (SLHNM25NK, Millipore, Darmstadt, Germany). For the lentiviral transduction, 2 × 10^4^ cells were plated on a feeder-covered 12-well plate per well. Then, the virus particles and fresh media were added at a ratio of 1:1, supplemented with 4 µg/mL polybrene for 12–16 h. The infected piPSCs were then plated on a feeder-coated 6-well plate after 12 h and cultured by the piPSCs culture medium for 5–7 days. After 1 week, stably-infected colonies were screened with puromycin (10 µg/mL) for 24 h, and then the cell lines were successfully established.

### 4.4. AP Staining

Cells were fixed with 4% paraformaldehyde in PBS (pH 7.4) for 15 min at room temperature, washed twice using ice-cold PBS, and developed with AST Fast Red TR and α-Naphthol AS-MX Phosphate (N4875, Sigma-Aldrich, Shanghai, China) according to the instructions [35,36,37]. The cells were incubated with the mixture (1.0 mg/mL Fast Red TR and 0.4 mg/mL Naphthol AS-MX in 0.1 mol/L Tris-HCL Buffer) at room temperature for 20 min. The AP-positive colonies were then shown in red. The images were captured by a Nikon phase-contrast microscope.

### 4.5. RNA Extraction, Reverse Transcription and qRT-PCR Detection

Total RNA was extracted by using an RNAiso Plus reagent (AL11817A, TaKaRa, Beijing, China) according to protocol. The total RNA extracted from CHIR- and sh-AXIN2CHIR- piPSCs were collected after the CHIR99021 withdrawal for three passages, and the total RNA extracted from sh-AXIN1, sh-AXIN2, OE-AXIN1, and OE-AXIN2 ipPSCs were collected after cell line purification. Then, by using the PrimeSript™ RT reagent Kit (Tiangen, China) according to its protocol, the cDNA was synthesized as a reverse transcription PCR (RT-PCR). The qRT-PCR reaction system was 20 μL in volume: 10 μL SYBR^®^ Premix Ex Taq II (Vazyme), 0.5 μL cDNA, 0.4 μL PCR Forward Primer (10 μM), 0.4 μL PCR Reverse Primer (10 μM), and RNase-free water added to a total volume of 20 μL.

### 4.6. Western Blot

The cells were lysed by cold RIPA buffer (Beyotime, Shanghai, China) for 30 min on ice, added to 5× SDS-PAGE loading buffer (Beyotime), and heated at 100 °C for 10 min. The lysates were then separated by 8–12% SDS-PAGE and transferred electrophoretically onto polyvinylidene difluoride (PVDF) membranes by Trans-Blot SD Cell and Systems (Bio-Rad) for 45 min at 15 V. After blocked with 5% non-fat milk in TBS-T buffer (20 mM Tris/HCl pH 8.0, 150 mM NaCl, 0.05% Tween 20), PVDF membranes were incubated with the primary antibodies [34]. After incubation with a secondary antibody, the signals were measured using a chemiluminescent imaging system (Tanon, Shanghai, China). Beta-ACTIN was used as an endogenous loading control. Antibodies used in this study were: Cyclin D1 (WL01435a, Wanleibio), *CASPASE3* (WL02117, Wanleibio), β-Catenin (51067-2-AP, Proteintech, Chicago, IL, USA) AXIN1(16541-1-AP, Proteintech), AXIN2(20540-1-AP, Proteintech) and β-Actin (AC028, ABclonal, Wuhan, China).

### 4.7. Apoptosis Detection

The apoptosis detection was performed by the Annexin V-FITC/PI Apoptosis Detection Kit (Vazyme, A211) according to the manufacturer’s instructions [29]. In brief, the cell suspension was washed twice with pre-cooled PBS and centrifuged at 1000× *g* for 5 min. We added 100 µL 1× Binding Buffer and blowed gently to make a single-cell suspension. We then added 5 µL Annexin V-FITC and 5 µL PI Staining Solution and incubated for 10 min at room temperature. Finally, we added 400 µL 1× Binding Buffer and mixed gently. Then, the images were captured with the phase-contrast microscope (Nikon, Tokyo, Japan).

### 4.8. RNA-Seq Analysis

Total RNA of the RNA samples was isolated from cell pellets by a Trizol reagent (TaKaRa), and the RNA-Seq library was generated according to the manufacturer’s recommendations [32]. Paired-end 150-bp sequencing was performed on an Illumina HiSeq2500 PE150 by SEQHEALTH Technology Corporation (China). Clean reads were aligned to the pig genome Ssc11.1, and the expression level was normalized as RPKM with a gene annotation file. Differential expression genes and functional enrichment for Gene Ontology (GO) and KEGG were performed with the GO stats package.

### 4.9. Immunofluorescence Staining

The cells were fixed with 4% paraformaldehyde in PBS (pH 7.4) at room temperature for 10 min [38]. Fixed cells were washed three times using ice-cold PBS, permeabilized with 0.1% Triton X-100 in PBS for 10 min, and then subsequently blocked for 1 h at room temperature in PBS containing 10% FBS. The cells were incubated with a blocking buffer containing CTNNB1 antibodies (51067-2-AP, Proteintech) at 4 °C overnight. The Alexa Fluor^®^ 594 conjugate goat anti-rabbit IgG (H + L; #ZF-0516; ZSGB-BIO) was diluted in a blocking buffer and incubated at room temperature for 1 h. After washing with PBS three times, the nuclei were stained with 10 µg/mL DAPI for 10 min. Finally, the images were collected by an EVOS fluorescence microscope.

### 4.10. Bimolecular Fluorescence Complementation

*CTNNB1* was PCR-amplified from piPSCs and were then subcloned into pBiFC-VC155 vectors [39]. *TCF3* and *TCF5* were PCR-amplified from piPSCs and were then subcloned into pBiFC-VN173 vectors. HEK293T cells were seeded onto a 6-well plate and grown to 70–80% confluence. Then, vectors were transfected into HEK293T cells using PEI (polyethyleneimine, Sigma) according to the manufacturer’s instructions. There were three groups: HEK293T cells with VN173TCF3-VC155CTNNB1, HEK293T cells with VN173TCF5- VC155CTNNB1, and HEK293T cells with VN173-VC155 as a control (NC).

## 5. Conclusions

In summary, our study suggested that porcine AXIN2 exhibits a stronger effect than AXIN1 on piPSCs, which affects the proliferation and survivability of piPSCs. Our study also revealed the potential mechanism underlying this process.

## Data Availability

The raw sequencing reads of this study have been deposited in the NCBI Sequence Read Archive (SRA) database under PRJNA772804.

## Supplementary Materials

The following are available online at https://www.mdpi.com/article/10.3390/ijms222312954/s1.
