# Supplementary material for: AXIN2 Reduces the Survival of Porcine Induced Pluripotent Stem Cells (piPSCs)
Source: Int J Mol Sci. 2021 Nov 30;22(23):12954. doi: 10.3390/ijms222312954 (PMC8658036; doi:10.3390/ijms222312954)
Supplement: Supplementary file 1 [file ijms-22-12954-s001.zip › ijms-1457446-supplementary.pdf]

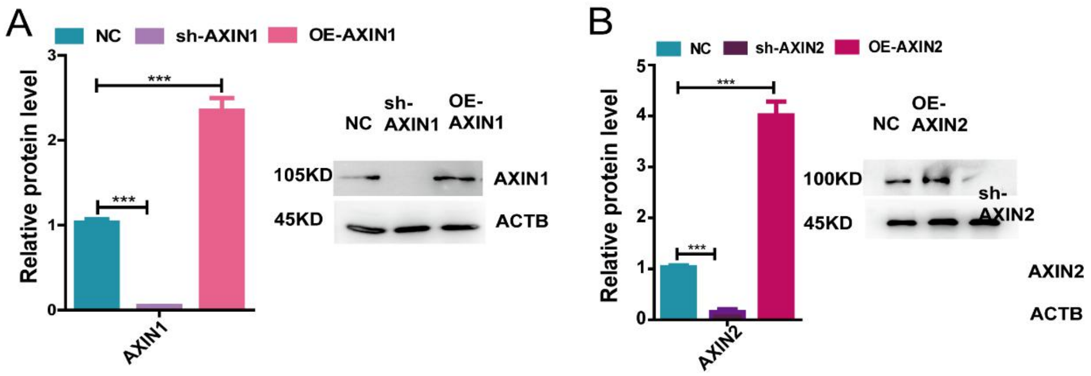

Supplementary Figure S1. Western blotting analysis of AXIN1 and AXIN2.

A, Western blot analysis of AXIN1 in the NC, sh-AXIN1 and OE-AXIN1groups; B Western blot analysis of AXIN2 in the NC, sh-AXIN2, and OE-AXIN2 groups; *** represent $P < 0.0001$.

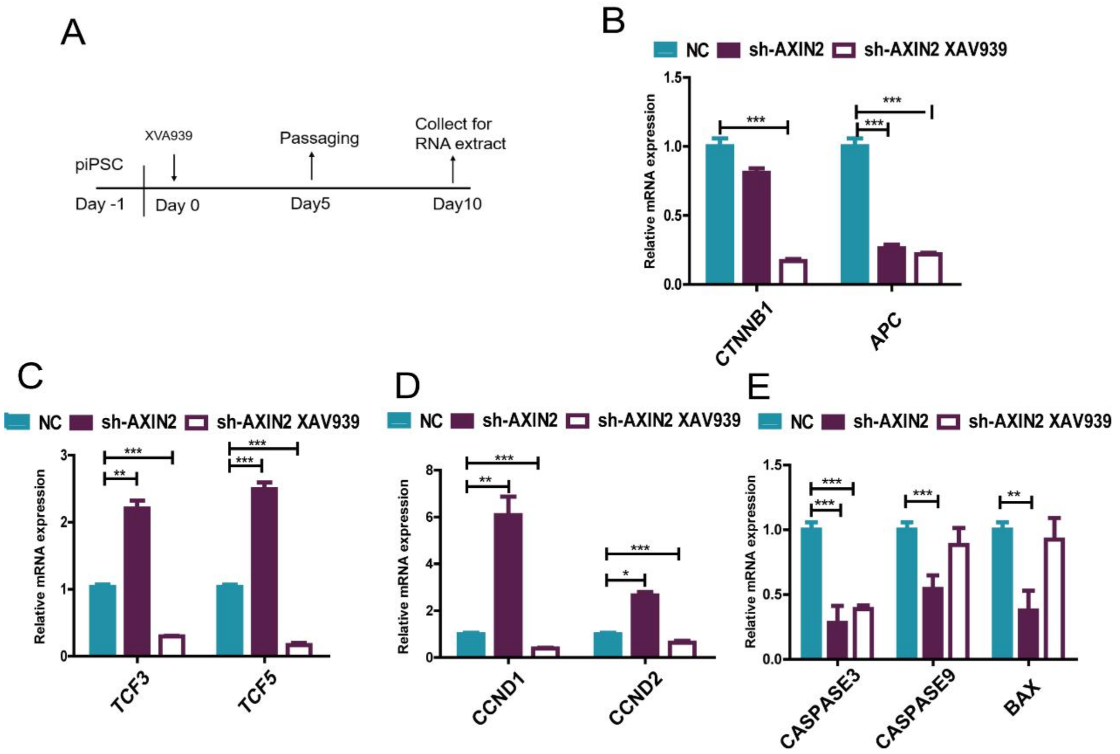

Supplementary Figure S2. CTNNB1 regulated the proliferation of piPSCs. A, A schematic illustration of the experiment; B, qPCR analysis of the expression levels of *CTNNB1* and *APC* in the control (NC), knockdown AXIN2(sh-AXIN2) and knockdown AXIN2 with XAV939 (sh-AXIN2 XAV939) groups; C, qPCR analysis of the expression levels of *TCF3, TCF5* in NC, sh-AXIN2 and sh-AXIN2 XAV939 groups; D, qPCR analysis of the expression levels of *CCND1, CCND2* in NC, sh-AXIN2 and sh-AXIN2 XAV939 groups; E, qPCR analysis of the expression levels of *CASPASE3*, CASPASE9 and BAX in NC, sh-AXIN2 and sh-AXIN2 XAV939groups; * represent $P < 0.05$; ** represent $P < 0.01$; *** represent $P < 0.0001$.
